# Iron Oxide and Titanium Dioxide Nanoparticle Effects on Plant Performance and Root Associated Microbes

**DOI:** 10.3390/ijms161023630

**Published:** 2015-10-05

**Authors:** David J. Burke, Nicole Pietrasiak, Shu F. Situ, Eric C. Abenojar, Mya Porche, Pawel Kraj, Yutthana Lakliang, Anna Cristina S. Samia

**Affiliations:** 1 Holden Arboretum, Kirtland, OH 44094, USA; E-Mail: npietrasiak@jcu.edu; 2 Department of Chemistry, Case Western Reserve University, Cleveland, OH 44106, USA; E-Mails: shu.situ@case.edu (S.F.S.); eca20@case.edu (E.C.A.); mya.porche@case.edu (M.P.); Pawel.Kraj@live.mercer.edu (P.K.); Yutthana.Lakliang@case.edu (Y.L.); 3 Department of Chemistry, Mercer University, Macon, GA 31207, USA

**Keywords:** iron oxide nanoparticles, titanium dioxide nanoparticles, arbuscular mycorrhizal fungi, *Glycine max*, microbial communities, nitrogen-fixing bacteria

## Abstract

In this study, we investigated the effect of positively and negatively charged Fe_3_O_4_ and TiO_2_ nanoparticles (NPs) on the growth of soybean plants (*Glycine max.*) and their root associated soil microbes. Soybean plants were grown in a greenhouse for six weeks after application of different amounts of NPs, and plant growth and nutrient content were examined. Roots were analyzed for colonization by arbuscular mycorrhizal (AM) fungi and nodule-forming nitrogen fixing bacteria using DNA-based techniques. We found that plant growth was significantly lower with the application of TiO_2_ as compared to Fe_3_O_4_ NPs. The leaf carbon was also marginally significant lower in plants treated with TiO_2_ NPs; however, leaf phosphorus was reduced in plants treated with Fe_3_O_4_. We found no effects of NP type, concentration, or charge on the community structure of either rhizobia or AM fungi colonizing plant roots. However, the charge of the Fe_3_O_4_ NPs affected both colonization of the root system by rhizobia as well as leaf phosphorus content. Our results indicate that the type of NP can affect plant growth and nutrient content in an agriculturally important crop species, and that the charge of these particles influences the colonization of the root system by nitrogen-fixing bacteria.

## 1. Introduction

Metal oxide nanoparticles (NPs) are being increasingly used for commercial applications ranging from inclusion in self-cleaning coatings, topical sunscreens, and antimicrobial soaps [1,2,3,4,5]. With the large production of NPs for use in everyday consumer products, NP contamination in the environment is becoming an important matter of concern [3]. Studies have shown that these materials can have significant negative effects on both plants and soil microbes in agro-ecosystems but the effects of specific metal oxide NP types and the conditions under which negative effects may be observed have still not been well characterized [1,6,7]. Titanium dioxide (TiO_2_) NPs, for example, produce reactive oxygen species when exposed to biological organisms or ultraviolet light. The reactive oxygen species can damage cell structures and DNA [8] and some studies have found negative effects of TiO_2_ NPs on plant growth (e.g., reduced cell elongation, reduced transpiration and leaf growth) [8,9]. TiO_2_ NPs have also been shown to change the communities of soil bacteria and reduce the prevalence of some taxa involved in nitrogen fixation (e.g., *Bradyrhizobium*) [10]. Communities of important plant symbionts, such as arbuscular mycorrhizal (AM) fungi that colonize plant roots and enhance nutrient uptake have also been found to be affected by the application of TiO_2_ NPs [11], which could have significant long-term effects on plant nutrient uptake and productivity in agro-ecosystems. On the other hand, some studies have reported no negative effects of TiO_2_ NPs on plant growth, especially at low concentrations, as compared to other NPs such as Ag and ZnO NPs that had strong negative effects on plant growth including reduced root growth and elongation [12,13]. Although well studied to date, the effect of TiO_2_ NPs on plants is still not thoroughly understood.

Results of studies with other metal oxide NP types have observed greater negative effects on plant and microbial systems as compared to TiO_2_. For example, ZnO NPs have been the focus of a number of studies and generally, the application of ZnO has negative effects on plants. Boonyanitipong *et al.*, found that the growth and elongation of rice roots were negatively affected by ZnO [12] and Lin and Xing found that ZnO NPs led to detrimental root growth termination of test plants [14]. Furthermore, the exposure of soybean plants to ZnO NPs led to a decrease in above ground and root mass, and altered the soil bacterial communities [15,16]. On the other hand, Ag NPs have also been found to be particularly toxic even at low concentrations. Kim *et al.* discovered that duckweed (*Lemna paucicostata*) cells experience the toxic effects of Ag NPs even at low 1 ppm concentrations, whereas toxic effects of TiO_2_ NPs were not evident until concentrations >250 ppm [13]. Recent reports have indicated negative and positive effects of different types of NPs on plants in terms of their growth and seed germination [17]. Several factors have been shown to influence the plant-NP interactions, including plant type, growth media, as well as the NP concentration, size, and surface area. To date, there is still no conclusive explanation and definite mechanism on the toxicity of NPs in plants, particularly in the soil environment, and further studies are needed to explore the effects of NPs in relation to plant growth [17,18].

NPs can alter soil microbial communities especially the microbes within the rhizosphere, which is the area of the soil in close proximity to the root that is under the influence of root exudates. Since plants rely upon these soil microbes for nutrient uptake (as is the case with AM fungi) or in the cycling and availability of nutrients like nitrogen (N) or phosphorus (P), changes in these soil microbes can have large effects on plants growth. As noted above, radical oxygen species liberated by NPs can damage cells and DNA, and some NPs can release heavy metals that can have toxic effects on microbial cells [19]. For example, the release of silver from NPs can inactivate enzymes, interfere with DNA replication, and alter cell membrane permeability, all of which can reduce microbial growth or have lethal effects on soil microbes. Although some NPs may be too large for microbes to absorb and assimilate, they can bind to the surface of cell membranes, altering membrane integrity and function. Various studies have demonstrated the toxic effects of different NPs on microbes in both the laboratory and field settings. Emami-Karvani and Chehrazi found that ZnO NPs affected gram positive bacteria more than gram negative bacteria, and that smaller sized NPs had greater antimicrobial effects [20]. Silver NPs are reported to be toxic to ammonifying and nitrogen fixing bacteria [6,21], and may disrupt microbial denitrification [6,21] and nitrogen-fixation [7]. Effects on the functional properties of soil microbes will depend on the composition and morphology of the NP under consideration and its local concentration. For example, Priester *et al.* found that ZnO had relatively little effect on nitrogen-fixation in a soybean crop system, but CeO_2_ had significant negative effects on nitrogen fixation at medium and high concentrations [7]. These studies suggest that toxic effects on soil microbes could be selective, and inhibition of certain microbial groups could alter soil microbial communities within the plant rhizosphere with negative consequences for plant nutrient uptake and soil fertility.

Although comparative studies on the effects of NPs have been performed, most studies are in culture or conducted under artificial conditions, and their applicability to plant-microbial responses in agro-ecosystems is uncertain. The work by Priester *et al.* demonstrates that additional comparative work, which simultaneously examines plant, microbial, and microbial functional processes in soil that underlie fertility, is greatly needed [7]. In this study, we report the results of a greenhouse experiment testing the effects of two important metal oxide NPs, Fe_3_O_4_ and TiO_2_, in a plant soil system using soybean (*Glycine max*.) as the test plant species. Fe_3_O_4_ is understudied within plant-soil systems and we wanted to examine the effects of this NP on plant growth and microbial communities in comparison to a better studied TiO_2_ NP system. We also examined the effects of different NP charges on the plant-soil system and the effects of NP concentration. We hypothesized that (1) treatment of soil with Fe_3_O_4_ would have fewer effects on plant growth and nutrient content of soybean compared to TiO_2_; (2) that both Fe_3_O_4_ and TiO_2_ would alter communities of microbes that grow in close association with plants (*i.e.*, microbial symbionts) such as nitrogen-fixing bacteria (*i.e*., rhizobia) as well as AM fungi; and (3) that the concentration and charge of the NP would affect plant uptake of NP in roots and translocation to above ground plant tissue such as leaves and stems. In our study, the plant growth response was assessed by whole plant biomass and nutrient analysis, while root microbial community responses were determined using DNA-based methods or biomass measurements as needed.

## 2. Results and Discussion

### 2.1. Nanoparticle Synthesis and Surface Functionalization

**Figure 1:**
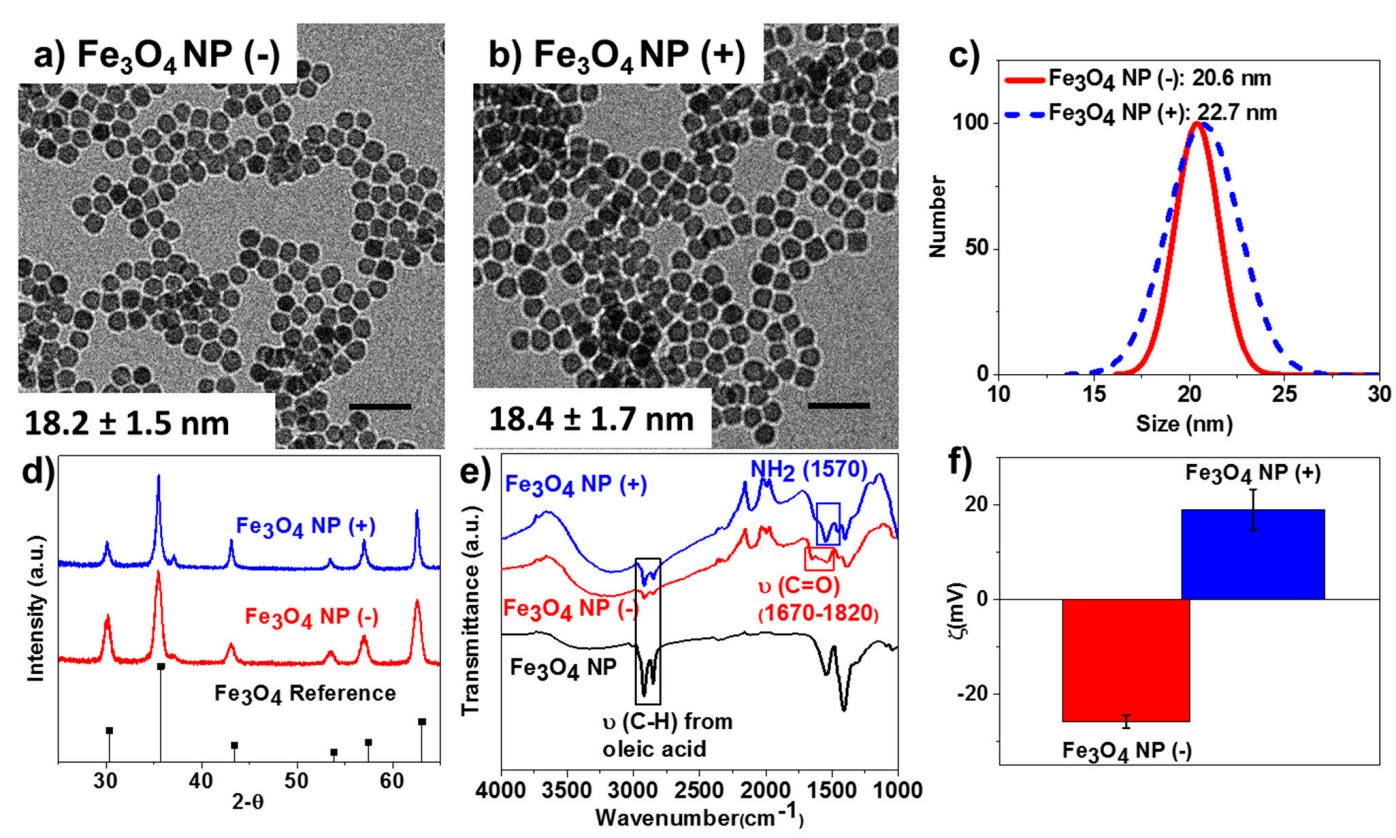
(**a**,**b**) Transmission electron micrographs of magnetite nanoparticles (NPs) (Fe_3_O_4_) functionalized with COOH (−) and NH_2_ (+) groups, respectively. Scale bars are 50 nm; (**c**) Dynamic light scattering measurements; (**d**) powder X-ray diffraction patterns; (**e**) Fourier transform infrared (FTIR) spectra; and (**f**) zeta potential measurements of the synthesized Fe_3_O_4_ NP (−) and Fe_3_O_4_ NP (+) samples, respectively.

Iron oxide NPs were synthesized according to a previous reported procedure [22]. Monodisperse and spherical Fe_3_O_4_ NPs were prepared via a one-step thermal decomposition method. The average diameter of the Fe_3_O_4_ NPs was evaluated to be 18 nm from transmission electron microscopy (TEM) analysis. The as-synthesized Fe_3_O_4_ NPs were capped with oleic acid and were later functionalized with amine- or carboxylic acid-terminating ligands through a silane ligand-based exchange water phase transfer process. The surface modification process yielded monodisperse Fe_3_O_4_ NPs with uniform shape and size (Figure 1a,b) and average hydrodynamic radius in the range of 21 to 23 nm as evaluated by dynamic light scattering (DLS) measurements (Figure 1c). The crystalline phase of the functionalized Fe_3_O_4_ NPs was confirmed to be magnetite (Fe_3_O_4_) through powder X-ray diffractometry (XRD) and Rietveld analysis (Figure 1d). The Fourier transform infrared (FTIR) spectra of the functionalized Fe_3_O_4_ NPs showed the NH_2_ (1570 cm^−1^) and C=O (1670–1820 cm^−1^) vibrational stretch peaks characteristic of amine- and carboxylic acid-functional groups (Figure 1e). The resulting amine- and carboxylic acid-functionalized Fe_3_O_4_ NPs carried positive and negative surface charges, respectively, as confirmed from zeta potential measurements (Figure 1f).

**Figure 2:**
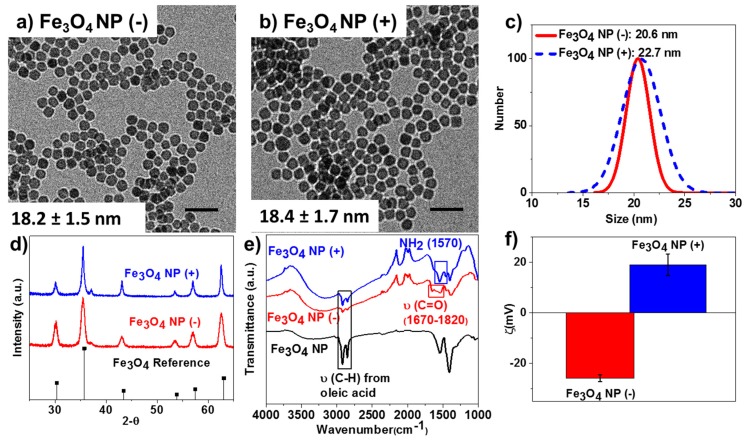
(**a**,**b**) Transmission electron micrographs of Degussa P25 (TiO_2_) functionalized with COOH (−) and NH_2_ (+) groups, respectively. Scale bars are 50 nm; (**c**) dynamic light scattering measurements; (**d**) powder X-ray diffraction patterns; (**e**) FTIR spectra; and (**f**) zeta potential measurements of TiO_2_ P25 (−) and TiO_2_ P25 (+) samples, respectively.

On the other hand, commercially available TiO_2_ Degussa P25 NPs (Evonik) were similarly modified with amine and carboxylic acid based silane ligands to generate positive and negatively charged TiO_2_ NPs. The size, morphology, and crystalline phase of the NPs were investigated using TEM, DLS, and powder XRD. The TiO_2_ P25 NPs have particle sizes in the range of 22 and 25 nm based on TEM analyses (Figure 2a,b), while DLS measurements indicated the formation of large aggregates in the size range of 147 to 340 nm for the TiO_2_ NPs in solution (Figure 2c). XRD analyses showed the TiO_2_ NPs indexing well to the crystalline anatase phase as well as that of rutile (Figure 2d). FTIR analysis showed the successful surface modification of the NPs (Figure 2e) while zeta potential measurements confirmed the surface charge of the NPs (Figure 2f).

### 2.2. Plant Growth and Nutrient Analysis

**Figure 3:**
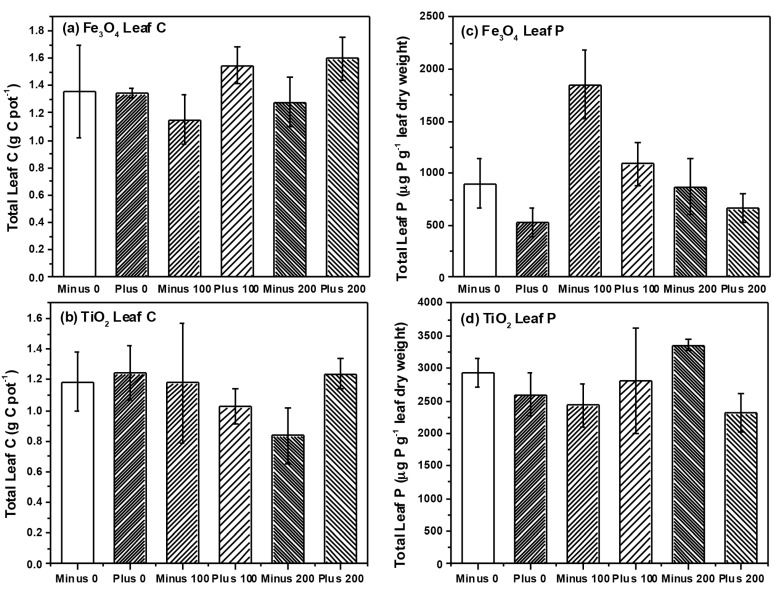
The carbon contents found in the leaf from soybean plants treated with (**a**) Fe_3_O_4_ and (**b**) TiO_2_ NPs, respectively; the phosphorus contents found in the leaf from soybean plants treated with (**c**) Fe_3_O_4_ and (**d**) TiO_2_ NPs, respectively.

Above ground dry biomass was significantly affected by NP type (three-way ANOVA, *F* = 6.5, *p* = 0.013) with plants from pots treated with Fe_3_O_4_ NPs having significantly greater dry biomass than plants treated with TiO_2_ NPs (3.29 ± 0.16 g·pot^−1^ for Fe_3_O_4_ *versus* 2.69 ± 0.18 g·pot^−1^ for TiO_2_) (Table 1). Root dry biomass was also marginally significant higher (three-way ANOVA, *F* = 3.2, *p* = 0.078) in plants treated with Fe_3_O_4_ (0.64 ± 0.04 g·pot^−1^ for Fe_3_O_4_ *versus* 0.54 ± 0.03 g·pot^−1^ for TiO_2_). Although leaf N content was unaffected by treatment, leaf C content was marginally significant higher (three-way ANOVA, *F* = 3.4, *p* = 0.069) in Fe_3_O_4_ treated as compared to TiO_2_ treated plants (43.02 ± 0.11 g·pot^−1^ for Fe_3_O_4_ *versus* 42.63 ± 0.19 g·pot^−1^ for TiO_2_) (Table 1, Figure 3a,b). Charge and NP concentration had no significant effect on above ground dry biomass, root dry biomass, or leaf C and N content. We found that both NP type and charge had significant effects on leaf P content (three-way ANOVA, *F* = 84.3, *p* < 0.001 NP type; *F* = 4.2, *p* = 0.047 NP charge). Leaf P was greater in TiO_2_ treatments as compared to Fe_3_O_4_ treatments (2738.8 ± 144.0 μg·P·g^−1^ leaf dry weight for TiO_2_ *versus* 984.1 ± 125.8 μg·P·g^−1^ leaf dry weight for Fe_3_O_4_) and leaf P was greater in plants that received negatively charged NPs (2056.7 ± 145.4 μg·P·g^−1^ leaf dry weight for negative *versus* 1666.2 ± 124.0 μg·P·g^−1^ leaf dry weight for positive) (Table 1, Figure 3c,d). We also saw a marginally significant metal *×* concentration effect on leaf P (Table 1). Further, two-way ANOVA of NP type performed separately (Table 2) found that within NP type (*i.e.*, Fe_3_O_4_ or TiO_2_), plant dry biomass and leaf C and N content were not affected by NP concentration or charge. However, leaf P content was significantly affected by both concentration and charge within the Fe_3_O_4_ treatments. Leaf P content in control and plants exposed to 200 mg of Fe_3_O_4_ NPs had approximately equivalent leaf P content but leaf P content was significantly higher in plants exposed to 100 mg of Fe_3_O_4_ NPs (715.4 ± 153.8 μg·P·g^−1^ leaf dry weight, control leaf P; 1470.7 ± 153.8 μg·P·g^−1^ leaf dry weight for 100 mg Fe_3_O_4_ NP; 766.1 ± 144.3 μg·P·g^−1^ leaf dry weight for 200 mg Fe_3_O_4_ NP) (Figure 3c,d). Plants receiving negatively charged Fe_3_O_4_ NPs also had significantly higher leaf P content than did plants receiving positively charged Fe_3_O_4_ NPs (1207.5 ± 132.9 μg·P·g^−1^ leaf dry weight, negatively charged Fe_3_O_4_; 760.6 ± 116.0 μg·P·g^−1^ leaf dry weight, positively charged Fe_3_O_4_) (Table 2, Figure 3c,d).

**Table 1:** Results of three-way ANOVA using the general linear model for effects of nanoparticle concentration and charge on plant growth performance. *p* values are shown where significant differences (*p* < 0.1) are in bold. Note: nutrient contents refer to leaf tissue only. (Above Wt = weight of plant tissue above ground, Root Wt = weight of roots, Nodule Wt = weight of nodule, Nodule/Root = nodule weight per root mass, AM Col = arbuscular mycorrhizal colonization).

Source of Variation	Above Wt ^1^	Root Wt	Nodule Wt	Nodule/Root ^2^	AM Col ^3^	C ^4^	N	P
Metal Type	**0.013**	**0.078**	0.222	0.866	0.199	**0.069**	0.808	**<0.001**
Metal Concentration	0.730	0.618	0.750	0.157	0.750	0.465	0.662	0.370
Metal Charge	0.373	0.228	**0.082**	**0.073**	0.878	0.110	0.938	**0.047**
Metal *×* Concentration	0.587	0.590	0.722	0.421	0.163	0.240	0.641	**0.093**
Metal *×* Charge	0.591	0.945	0.777	0.511	0.245	0.242	0.327	0.769
Concentration *×* Charge	0.182	0.317	0.396	0.559	0.171	0.194	0.570	0.665
Metal *×* Conc. *×* charge	0.100	0.972	0.322	0.225	0.630	0.234	0.977	0.134

^1^ Plant tissue and nodule weight are reported on a dry weight basis per pot; ^2^ nodule colonization: nodule dry weight/root dry weight; ^3^ AM colonization: copy numbers of 18S rDNA per gram of dry root tissue. Data was natural log transformed to meet normality assumptions; ^4^ C and N data are % C and N leaf data log transformed prior to analysis.

**Table 2:** Results of two-way ANOVA using the general linear model for effects of nanoparticle concentration and charge on plant growth performance. *p* values are shown where significant differences (*p* < 0.1) are in bold. Note: nutrient contents refer to leaf tissue only.

Source of Variation	Above Wt ^1^	Root Wt	Nodule Wt	Nodule/Root ^2^	AM Col ^3^	C ^4^	N	P
Fe_3_O_4_ Concentration	0.772	0.647	0.749	0.257	0.842	0.528	0.778	**0.003**
Fe_3_O_4_ Charge	0.284	0.494	0.152	**0.057**	0.188	**0.010**	0.387	**0.018**
Concentration *×* Charge	0.138	0.590	0.307	0.258	0.604	0.887	0.507	0.442
TiO_2_ Concentration	0.578	0.521	0.719	0.267	0.156	0.297	0.631	0.899
TiO_2_ Charge	0.814	0.252	0.309	0.462	0.412	0.805	0.547	0.377
Concentration *×* Charge	0.159	0.475	0.441	0.460	0.479	0.157	0.863	0.350

^1^ Plant tissue and nodule weight are reported on a dry weight basis per pot; ^2^ nodule colonization: nodule dry weight/root dry weight; ^3^ AM colonization: copy numbers of 18S rDNA per gram of dry root tissue. Data was natural log transformed to meet normality assumptions; ^4^ C and N data are % C and N leaf data log transformed prior to analysis.

### 2.3. Analysis of Microbial Communities

We detected a total of 15 TRFs in our root samples representing AM taxa. Non-metric multidimensional scaling (NMS) analysis resulted in a final stress of 10.1 and a three dimensional solution. However, we found no effect of NP type (*i.e.*, Fe_3_O_4_ or TiO_2_), concentration or charge on AM root communities (data not shown). Multi-response permutation procedures (MRPP) analysis confirmed a lack of treatment effects on the AM root communities. Our analysis also failed to find any effects of NP type, charge or concentration on AM root colonization (Table 1 and Table 2), although AM colonization tended to increase with Fe_3_O_4_ NP concentration but decrease with TiO_2_ NP concentration. We also analyzed a subset (*n* = 37 plants) of root nodules for differences in rhizobia communities using NMS and MRPP. NMS resulted in a two-dimensional solution with a final stress of 3.9 and we were able to detect five different rhizobia taxa within the roots of soybean. However, we found no effects of NP type, concentration or charge on rhizobia communities using NMS and confirmed through MRPP (Data not shown). We did, however, find a marginally significant effect of NP charge on nodule dry weight and nodule root colonization (three-way ANOVA, *F* = 3.1, *p* = 0.082 nodule dry weight; *F* = 3.3, *p* = 0.073 NP nodule colonization) (Table 1). Two-way ANOVA found that nodule colonization was significantly higher in plants that received positively charged Fe_3_O_4_ NPs (0.337 ± 0.02 g dry weight per gram root dry weight, positively charged Fe_3_O_4_; 0.275 ± 0.02 g per gram root dry weight, negatively charged Fe_3_O_4_) but application of TiO_2_ NPs had no effect on nodule colonization.

**Figure 4:**
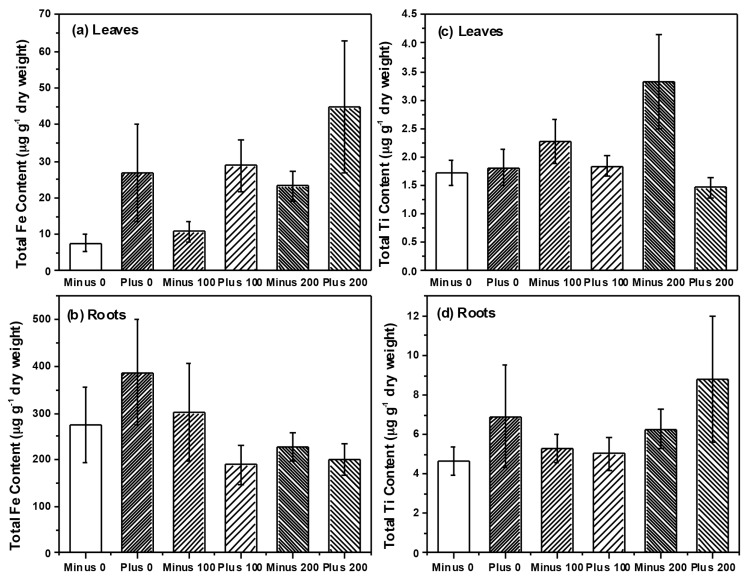
Total amount of Fe found in leaves (**a**) and roots (**b**) and the total amount of Ti found in leaves (**c**) and roots (**d**), respectively.

The metal oxide NP type had different effects on plant growth, nutrient uptake, and colonization by soil microbes. To better examine the phytotoxicity effect of soil contaminated by NPs, we have chosen to grow the soybean plants in soil with TiO_2_ and Fe_3_O_4_ NPs treatments that were applied as water suspension to mimic the condition of soil contamination by NP run offs. Such an application method has been commonly used in previous soil-based plant studies and has provided sufficient exposure and bioavailability of the NPs to the plants [7,23,24]. We found that TiO_2_ NPs significantly reduced plant growth as compared to Fe_3_O_4_ and these changes were also accompanied by reductions in leaf C content. NP types have been found to differentially affect plant growth in previous studies. For example, Priester *et al.* found that CeO_2_ NPs reduced plant growth and impaired N-fixing ability of soybean while ZnO NPs did not affect growth (although ZnO NPs did accumulate in above ground plant tissue) [7]. We previously found no significant effects of TiO_2_ NPs on plant growth at similar concentrations to those used in the current study [11], and dry biomass was also similar after a comparable growth period (e.g., 2.94 ± 0.21 g·pot^−1^ TiO_2_ treated above ground dry biomass) although root growth in our previous study was 2× lower than what we report here. It is possible that the significantly reduced dry biomass within the TiO_2_ NP treatment does not reflect a reduction in growth with exposure to TiO_2_, but rather stimulation in plant growth with exposure to Fe_3_O_4_. Several studies have reported positive effects of NP on plant growth. Hong *et al.* reported that spinach leaves treated with TiO_2_ NPs had higher levels of photosynthesis compared to untreated leaves, whereas Lu *et al.* reported increased levels of nitrate reductase activity in the rhizosphere of soybean roots treated with TiO_2_ NPs [25,26]. Studies of this type have suggested that treating plants with some NPs may improve plant growth and productivity [25,26,27,28]. Although we found no evidence for improved growth with TiO_2_ application, improved growth with Fe_3_O_4_ application could have resulted from changes in plant Fe status (Figure 4). Fe is a needed plant micronutrient [29], although it is usually not deficient except on calcareous soils. Increasing amounts of Fe_3_O_4_ could have had direct effects on plant growth increasing both above- and below-ground growth relative to TiO_2_ plants. It seems unlikely that growth increases would be due to changes in nutrient availability with NP application since leaf N was unaffected by NP type, concentration or charge, and leaf P was actually lower in Fe_3_O_4_ treated plants, where charge actually influenced leaf P content. Overall reductions in leaf P could be the result of Fe_3_O_4_ binding to soil P under acidic conditions, creating FePO_4_ complexes within the soil [30]. This could have reduced overall plant P acquisition relative to plants treated with TiO_2_ NP but growth increased despite these declines in leaf P (and no change in leaf N as noted above). Growth increases in the Fe_3_O_4_ plants was accompanied by marginally significant increases in leaf C, suggesting that increased C fixation could have contributed to the growth increases in the Fe_3_O_4_ plants despite leaf P reductions. Fe is necessary for the production of chlorophyll [31] and increased chlorophyll content could have led to greater C fixation and the growth increases we observed. More detailed analysis of plant photosynthesis and gas exchange would be needed to ascertain the cause of the growth increases with Fe_3_O_4_ application relative to TiO_2_.

As expected, we found that roots contained significantly higher levels of NP as compared to leaves, and this was the case for both NP examined (Table 3, Figure 4). Plants accumulated much more Fe than Ti (Figure 4), but given the fact that plants excrete organic compounds to solubilize and facilitate Fe uptake [31], high levels of Fe in root tissue, especially in comparison to Ti may not be unexpected. We detected very little transfer of Ti into the leaf tissue and there was no effect of charge or concentration on the leaf Ti levels. However, of interest were the effects of charge on Fe transfer into leaves. Although charge had no effect on root Fe concentrations, plants treated with negatively charged Fe_3_O_4_ NPs had significantly more Fe in leaf tissue (Table 3) than did plants treated with positively charged Fe_3_O_4_ NPs, suggesting that NP charge can affect the plant translocation of metals. ZnO NPs were found to be translocated by the plant into above ground plant tissue but CeO_2_ NPs were not [7], suggesting that NP type could also affect uptake and translocation. In our previous study, we found some evidence that doping TiO_2_ NPs with N could also affect plant translocation of NPs to above ground plant tissue [11]. Within our current study, we found that NP charge could also affect NP translocation. In addition, similar to the findings of other groups [32], the NP size also greatly influences the plant uptake. In our study, the TiO_2_ NPs formed large aggregates in the size range of 147–330 nm as evaluated from DLS analysis. In comparison, the Fe_3_O_4_ NPs that we used in our study have the hydrodynamic radius in the range of 22–25 nm. As a consequence of the large difference in size range, one can infer that the Fe_3_O_4_ NPs will be more likely to be available for the plant uptake because of its relatively larger surface area. This is reflected in our results where you can see a significant effect of NP concentration with the application of Fe_3_O_4_ NPs as compared to the plant exposed to TiO_2_ NPs. These collective data suggest that the plant uptake of NPs will be context specific, depending both on the type, size, charge, and chemical surface structure of the NP, but that all factors may affect uptake and translocation.

**Table 3:** Results of three-way ANOVA using the general linear model for effects of plant tissue (root or leaf), nanoparticle concentration, and nanoparticle charge on plant tissue metal concentrations. *p* values are shown where significant differences (*p* < 0.1) are in bold. Data for Fe_3_O_4_ were log transformed to meet normality assumptions.

Variable	Source of Variation	DF	SS	MS	*F*	*p*
Fe	Plant Tissue	1	24.306	24.306	216.747	<0.001
	Fe_3_O_4_ Concentration	2	0.138	0.0691	0.616	0.543
	Fe_3_O_4_ Charge	1	0.379	0.379	3.381	0.071
	Tissue *×* Concentration	2	0.624	0.312	2.783	0.070
	Tissue *×* Charge	1	0.496	0.496	4.424	0.040
	Concentration *×* Charge	2	0.293	0.147	1.308	0.278
	Tissue *×* Conc. *×* Charge	2	0.182	0.0911	0.812	0.449
	Residual	60	6.728	0.112		
	Total	71	33.148	0.467		
Ti	Plant Tissue	1	275.862	275.862	25.317	<0.001
	TiO_2_ Concentration	2	23.532	11.766	1.080	0.347
	TiO_2_ Charge	1	2.492	2.492	0.229	0.634
	Tissue *×* Concentration	2	10.868	5.434	0.499	0.610
	Tissue *×* Charge	1	20.869	20.869	1.915	0.172
	Concentration *×* Charge	2	6.466	3.233	0.297	0.744
	Tissue *×* Conc. *×* Charge	2	11.798	5.899	0.541	0.585
	Residual	55	599.305	10.896		
	Total	66	971.381	14.718		

**Figure 5:**
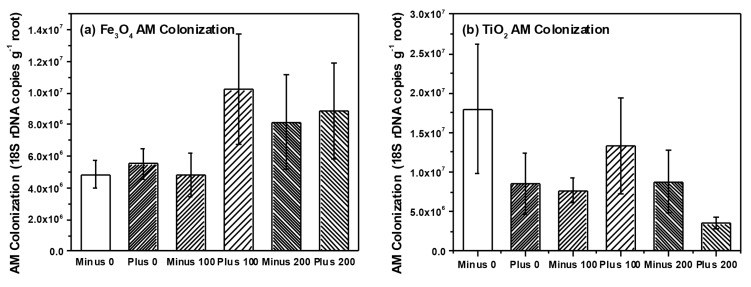
AM colonization in soybean plants treated with (**a**) Fe_3_O_4_ and (**b**) TiO_2_ NPs, respectively.

**Figure 6:**
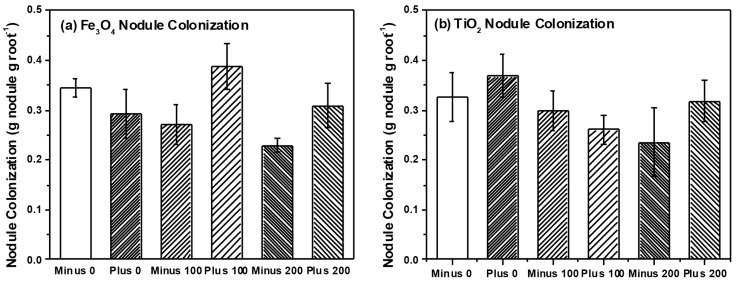
Nodule colonization of soybean plants treated with (**a**) Fe_3_O_4_ and (**b**) TiO_2_, respectively.

In our previous work, we found significant effects of TiO_2_ NPs on communities of AM fungi in soil surrounding plant roots (*i.e.*, rhizosphere soil) [11]. Although we found no effect on rhizosphere bacteria communities, our findings were in agreement with a number of studies that have observed effects of NP on soil microbes and their activity [7,10,11]. However, in our current study we specifically examined two groups of microbial plants mutualists and their colonization and community structure on or within the root tissue itself, and not in soil. We felt that by examining microbes colonizing roots, we would have a better sense of the functional effects of NPs (e.g., nutrient uptake, availability with rhizobia) than examination of soil communities only. Surprisingly, we found little effect on the community structure for either AM fungi (Figure 5) or for rhizobia (Figure 6) colonizing the root system, which is in contrast to our previous work and that of others [10,11]. However, bacteria and fungi within the root tissue could be protected from the direct toxic effects of NPs if the chemical form or concentration of the NP differs inside and outside the root. In our study, Fe concentration within the root ranged between 200–400 μg·Fe·g^−1^ root tissue while soil concentrations also ranged between 200–400 μg·Fe·g^−1^ soil. Consequently, the root analysis reflected the Fe concentration in the soil, while the Ti concentration within the roots were considerably less than the amount in the soil (200–400 μg·Ti·g^−1^ soil as compared to 5–10 μg·Ti·g^−1^ root tissue). Therefore, microbes within plant roots could have been protected against the toxic effects of TiO_2_ NPs since root concentrations were far less than in the soil, but this was not the case for Fe_3_O_4_ NP treatment. When examining colonization of roots directly, AM colonization tended to increase with Fe_3_O_4_ NP application and decrease with TiO_2_ NP application but these differences were not significant. In regards to rhizobia, we did observe a significant increase in nodule dry weight and colonization when exposed to positively charged Fe_3_O_4_ NP; a pattern not seen with TiO_2_ NP application (Figure 6). Although Priester *et al.* saw declines in N-fixation with CeO_2_ application, these declines were evident at only high concentrations and absent in ZnO application [7]. Lu *et al.* did report higher levels of nitrate reductase activity in the rhizosphere of soybean roots treated with TiO_2_ NPs, suggesting that NP could increase activity of some bacterial groups and have functional effects on soil nutrient availability [26]. Although we did not measure N-fixation *per se*, our results suggest that application of positively charged NPs could increase the rhizobia nodule dry weight per unit root weight of soybean roots, at least in comparison to the negatively charged Fe_3_O_4_ NPs. However, these results are only marginally significant and further study is necessary. One possible reason for the increase in rhizobia with Fe_3_O_4_ NP is that Fe is an essential micronutrient for nitrogen-fixing bacteria [33], and positively charged Fe_3_O_4_ NP could be providing these bacteria with an essential micronutrient, thus resulting in increased growth and colonization as we observed. We cannot discount, however, the possibility that positively charged Fe_3_O_4_ NPs adhere to the surface of the root nodules to a greater extent than negatively charged Fe_3_O_4_ NPs and this could explain the differences in the observed nodule dry weights.

## 3. Experimental Section

### 3.1. Materials

The ferroxyhydrate precursor [FeO(OH)], oleic acid (90%), 1-octadecene (90%), trimethylamine (99%), ammonium hydroxide (14.8 N), ascorbic acid (99%), ammonium molybdate, sodium arsenite, para-nitrophenol, toluene (HPLC grade 99%) and ethanol (HPLC grade 90%) were purchased from Sigma Aldrich (Milwaukee, WI, USA). Trichloroacetic acid, sodium citrate, acetic acid (99%), sulfuric acid, sodium hydroxide, and P standard were all purchased from Fisher Scientific (Pittsburgh, PA, USA). The silane functionalization agents (3-(triethyoxysilyl)propyl succinic anhydride (95%) and 3-(2-aminoethylamino)propyl trimethoxysilane (95%)) were purchased from Gelest (Philadelphia, PA, USA). Commercially available titanium dioxide (TiO_2_) Degussa P25 was purchased from Evonik Industries (Piscataway, NJ, USA). All chemicals and reagents were used as received.

### 3.2. Experimental Set-up and Plant/Soil Sampling

To examine plant and microbe response to NPs, we studied a widely planted agricultural crop species: *Glycine max* (L.) Merr. (soybean), under controlled greenhouse conditions. Seeds for soybean (variety Envy) were purchased from Johnny’s Selected Seeds (Fairfield, ME, USA). Seeds were planted into 15 cm plastic pots filled with 250 g of sterilized potting soil (Fafard, Agawam, MA, USA; 3B middle weight mix containing 50% sphagnum peat, processed pine bark, vermiculite, perlite, Dolomitic limestone, and starter nutrients), 200 g of sterile sand, and 50 g of air dried field soil for a total of 500 g of dry soil per pot. The potting soil and sand was sterilized by autoclaving for 40 min at 121 °C and was incorporated into the pots after the soil had cooled. Field soil served as a microbial inoculant for our study. The soil used for inoculating the greenhouse pots was collected from a fallow, former agricultural field located at the Holden Arboretum in northeast Ohio. The soil is silt loam topsoil currently occupied by a plant community consisting of non-native grasses and forbs including legumes. The soil pH at 10 cm depth was highly acidic with a pH of approximately 4.0, and the soil total carbon content is between 2%–3% while the soil total nitrogen content is between 0.1%–0.2%. Field soil was sieved to remove roots and extraneous material and well mixed before air drying. Field soil was incorporated into the pots containing the well mixed potting soil and sand. After pots were watered, three seeds of soybean were planted into each pot (72 pots total) on 13 January 2015 in a heated greenhouse and well-watered. Supplemental lighting was used and we maintained a 12-h day/night cycle that mimics spring growing conditions. Maximum greenhouse light levels were approximately 150 W·m^−2^ under overcast conditions while under bright sky conditions maximum greenhouse light levels were approached 550 W·m^−2^. Temperature was maintained between 24 and 29 °C and seedlings germinated and emerged within 10 days. After emergence, seedlings were thinned to one plant per pot.

On 14 January 2015, one day after planting seeds, we applied NPs to the pots. Treatments were randomly assigned to pots within each row but each treatment was represented within each plant row. In this way bench effects would not affect our results. Plants were rotated across the bench every week during the duration of the study to minimize location effects on plant growth. Pots received either 100 mL of distilled water and were designated as non-treated controls, or received 100 mL of distilled water with either 100 or 200 mg of synthesized Fe_3_O_4_ NP (−), Fe_3_O_4_ NP (+), TiO_2_ P25 (−) and TiO_2_ P25 (+). Shallow (2 cm deep) plastic trays were placed under each pot to catch any liquid that drained from the pot and prevent loss of nanoparticle during watering; any water that leaked from the pot was added back to the pot to maintain NP concentrations. Our experimental set-up allowed for a 2 × 2 × 3 factorial design to test for the effect of nanoparticle (Fe_3_O_4_ or TiO_2_-Degussa), charge (− or +) or concentration (0, 100, and 200 mg per 1 kg of soil) on plant growth and nutrient concentration as well as on microbial community composition within the plant root. Six replicate pots were established for each nanoparticle combination (2 NPs × 2 charges × 3 concentrations × 6 replicates = 72 pots). Plants were arranged on the greenhouse bench, where each row contained one replicate of each plant/nanoparticle treatment combination. Two weeks after seedlings emergence, plants were fertilized with 100 mL of half strength Peters water soluble general purpose fertilizer (20-20-20) (Scotts Company LLC, Marysville, OH, USA). This was the only supplemental fertilization applied during the study.

After about 6 weeks of plant growth (8 weeks from sowing), we began to harvest plant biomass and soil from the experimental pots. Since some plants grew faster than others, we harvested plants at the same phenological stage, as they began to set fruit. Plants were thus harvested sequentially, rather than at one time, so that all plants had reached the fruit set stage (R4 stage) and differences in biomass would not reflect differences in individual growth rates of the plants but would reflect treatment conditions. All above- and belowground biomass was collected and separated from the pots. Soil adhering tightly to fine roots (*i.e.*, rhizosphere soil) was gently shaken off, collected and retained. This soil was stored at −70 °C and for reserved for future analysis. A coarse mesh sieve (4 mm) was used to separate roots from the remaining soil through gentle washing with tap water. All fine roots recovered in this fashion were used to estimate below ground dry biomass. Root nodules formed by nitrogen-fixing bacteria were removed from roots and separately weighed; nodules were used to estimate colonization of roots by nitrogen fixing bacteria. A small portion of each plant root system was removed, separately weighed and used to estimate mycorrhizal root colonization and community structure.

### 3.3. Nanoparticle Synthesis and Characterization

#### 3.3.1. Synthesis of Magnetite Nanoparticles (Fe_3_O_4_ NPs)

Magnetite NPs were synthesized via a one-step thermal decomposition synthesis approach adopted and modified from the Colvin group [22]. In a typical large scale synthesis, 40 mmol of FeO(OH) fine powder was stirred with 150 mmol of oleic acid and 75 mL of 1-octadecene. The mixture was heated up to 320 °C and kept at reflux for 2 h. The NPs were separated from the reaction mixture via centrifugation after washing with toluene and ethanol. The as-synthesized iron oxide NPs were passivated by oleic acid and showed magnetite crystalline phase (Figure 1).

#### 3.3.2. Functionalization of Fe_3_O_4_ NPs to Introduce Surface Charges

The as-synthesized Fe_3_O_4_ NPs (1 g) were first dissolved in 500 mL toluene before mixing with 20 mL of trimethylamine and 200 mL of NH_4_OH in butanol (1 M). The dispersion was sonicated for 5 min before adding 5 mL of 3-(triethyoxysilyl)propyl succinic anhydride solution and 10 mL of water. This was further sonicated for 30 min. The NPs were kept at room temperature for 2 h until all of the NPs transferred in the water phase. The carboxylic group functionalized Fe_3_O_4_ NPs were separated through centrifugation and re-dispersed in water [34]. The final pH was adjusted to pH 7 using dilute acetic acid and triethylamine. For the introduction of amino groups, 1 g of as-synthesized Fe_3_O_4_ NPs was first dissolved in 500 mL toluene before mixing with 20 mL of trimethylamine. The dispersion was sonicated for 5 min before adding 5 mL of 3-(2-aminoethylamino)propyltrimethoxysilane solution. The mixture was stirred at room temperature for 48 h. The particles were then precipitated with petroleum ether and re-dispersed in ethanol. The precipitated particles were washed with ethanol and water a total of three times. The resulting amine functionalized NPs were re-dispersed in water and the pH was adjusted to 7 to ensure the presence of surface changes for both sample sets.

#### 3.3.3. Functionalization of Degussa TiO_2_ P25 NPs to Introduce Surface Charges

Commercially available titanium dioxide (TiO_2_) Degussa P25 (Evonik) was used and modified with amine and carboxylic acid-terminated ligands. Titania samples were dissolved in basic (pH = 8) aqueous solution and 3-(2-aminoethylamino)propyltrimethoxysilane was added and the solution was stirred vigorously and refluxed for 4 h to obtain amine-terminated titania sample. To obtain carboxylic acid terminated NPs, 3-(triethoxysilyl)propylsuccinic anhydride was used. The solutions were then centrifuged (8500 rpm, 30 min) and washed with water before dispersing in water (pH = 7).

#### 3.3.4. Characterization

A Tecnai T12 transmission electron microscope (TEM) operated at 80 kV was used to evaluate the size and morphology of the NPs. The software ImageJ was used to measure the particle size of an average of 200 particles to estimate the size distribution for each sample. The powder X-ray diffraction (PXRD) patterns of the samples were collected using a RigakuMiniFlex X-ray powder diffractometer using Cu Kα radiation (γ = 0.154 nm). The surface functionalization of the NPs were evaluated using attenuated total reflectance—Fourier transform infrared spectroscopy (ATR-FTIR) conducted in the range 600–4000 cm^−1^ using a Thermo Scientific Nexus 870 ATR-FTIR spectrometer. The particle size distributions were analyzed using dynamic light scattering (DLS) on a ZetaPALS particle size analyzer (Brookhaven, Upton, NY, USA) at a scattering angle of 90°. The elemental analyses were conducted using atomic absorption spectroscopy (AAS). UV-Visible absorption measurements were carried out using a CARY 50 BIO spectrophotometer. Standard solutions of 0, 0.1, 0.4, 1.0 and 2.0 ppm were prepared from a 10 ppm P standard stock solution. Calibration curves were generated using standards. Absorbance measurements were read using the peak at 700 nm.

### 3.4. Plant Growth and Elemental Analysis

Aboveground and belowground biomass harvested from the pots was dried at 60 °C for one week, and weighed. Once dry, all plant tissue, except for that portion of the root system reserved for DNA extraction, was finely ground in a tissue grinder to reduce particle size, and then pulverized in a Precellys homogenizer (Bertin Technologies, Montigny-le-Bretonneux, France) to produce a fine powder suitable for analysis of C and N. The total plant C and N were analyzed by a dry combustion method on an ECS 4010 CHNSO elemental analyzer (Costech Analytical, Valencia, CA, USA). Total leaf and root P was determined through acid digestion using sulfuric acid and 30% hydrogen peroxide [35] followed by colorimetric analysis using the modified ascorbic acid method [36]. All sample aliquots were adjusted to pH 8 before analysis. For total leaf and root Fe and Ti, samples were acid-digested at 150 °C for 4 h in pressurized Teflon lined vessels with 70% nitric acid. Samples were diluted to a final concentration of 3% nitric acid and analyzed for Fe using flame AAS. Samples were diluted to a final concentration of 2% nitric acid and analyzed for Ti using graphite furnace atomic absorption spectroscopy (GFAAS). A blank sample was run in between measurements to clean the pyrolytic graphite tube and eliminate Ti memory effects. The iron (Fe) standard solutions (0, 0.5, 1, 2, 4, 8 ppm) were prepared from a 1000 ppm in 3% HNO_3_ Fe stock solution (Ricca Chemical Co., Batesville, IN, USA). On the other hand, the titanium (Ti) standard solutions (0, 10, 20, 30, 40 ppb) were prepared from a 1000 ppm in 2% HNO_3_ Ti stock solution (Fluka Analytical, Sigma-Aldrich Co., St. Louis, MO, USA).

### 3.5. Analysis of Microbial Communities

DNA was extracted from wet roots using a bead beating protocol [37]. In short, 100–500 mg of roots were placed in a 1.5 mL bead beating tube containing 500 mg of sterile glass beads (300 mg of 400 µM glass beads (VWR, West Chester, PA, USA), 200 mg of 1 mm glass beads (Chemglass, Vineland, NJ, USA)) and 750 µL of 2% CTAB (cetyltrimethylammonium bromide). Samples were beaten for 90 s in a Precellys homogenizer at 6500 rpm. Approximately 500 mL of the supernatant was removed and purified by phenol/chloroform extraction and precipitation with 20% polyethylene glycol 8000 in 2.5 M NaCl [37]. DNA was suspended in 50 µL TE (Tris EDTA) buffer and stored at −20 °C until use.

For analysis of arbuscular mycorrhizal (AM) fungi, we targeted the 18S rRNA gene using AM specific primer AM1 [38] and general eukaryotic primer NS31 [39]. The PCR was conducted in 50 μL reaction volumes containing 1 μL of purified DNA (approximately 100 ng), 0.2 μM of each primer, 2.0 mM MgCl, 0.2 mM dNTP, 0.15 mg·mL^−1^ bovine serum albumin, and 2.0 units GoTaq DNA polymerase (Promega Corporation, Madison, WI, USA). The PCR was carried out on a PTC 100 Thermal Cycler (MJ Research, Boston, MA, USA) using cycling conditions in Helgason *et al.* except that 32 cycles were used for PCR and the annealing temperature was 60 °C [38]. The primer NS31 was labeled with the fluorochrome HEX (4,7,2ʹ,4′,5′,7′-hexachloro-6-carboxyfluorescein) and PCR product was used for analysis of AM fungal communities using terminal restriction fragment length polymorphism procedures [40]. PCR product was digested with the restriction enzyme *Hinf*I (Promega, Madison, WI, USA) and TRFLPs were completed through the Cornell Bioresource Center using an Applied BioSystems 3730xl DNA Analyzer and the GS600 LIZ size standard. Profiles were analyzed using Peak Scanner™ Software (version 1.0, Applied Biosystems 2006, Foster City, CA, USA) and only peaks that accounted for greater than 1% of the relative peak area were included in this analysis (*i.e.*, major TRFs) [41]. To estimate root length colonized by AM fungi, we used quantitative PCR (qPCR) using primer AM1 and primer AM1GF, which generates an approximately 210 base pair long amplicons [40]. The qPCR was conducted in 20 μL reaction volumes using 1× iTaqUniversal™ SYBR^®^ Green Supermix (Bio-Rad) on a MiniOpticon™ real-time PCR detection system (Bio-Rad Laboratories, Inc., Hercules, CA, USA) following procedures developed by Hewins *et al.* [40].Colonization is reported as copy number per gram of dry root tissue.

For analysis of nitrogen-fixing bacteria, we extracted DNA from root nodules using a modification of the procedure described above. In short, we bead beat nodules for 90 s, then ground the nodules within the tube with a sterile micro-pestle, before bead beating the nodules again for 45 s. Samples were purified by phenol-chloroform extraction and suspended in 100 µL TE buffer. To examine effects on nitrogen-fixing bacteria in root nodules, we targeted *nifH*, the structural gene for the enzyme nitrogenase reductase, using primers and conditions following Widmer *et al.* [42] Primer *nifH* (for Rev) was labeled with the fluorochrome HEX and used with primer *nifH* (for A). This generated an approximately 450 base pair PCR product that was used for TRFLP analysis using the procedure described above but using *Hae*III (Promega, Madison, WI, USA) as the restriction enzyme.

### 3.6. Data and Statistical Analysis

Differences in biomass, plant nutrient and metal content, and colonization by AM fungi and rhizobia were analyzed by 3-way ANOVA using procedures in SigmaStat 3.5 (Systat Software Inc., San Jose, CA, USA). The Kolmogorov-Smirnov test was used to determine if data were normally distributed prior to analysis. To examine uptake of metals (*i.e.*, Fe or Ti) we used 3-way ANOVA to determine effects of plant tissue, metal concentration and charge on metal distribution in plant tissue. Analysis was conducted on transformed (log 10) data for Fe in order for data to meet normality assumptions; Ti data met normality assumptions and analysis was conducted on untransformed data. Since 3-way ANOVA showed differences in how plants responded to metal type (*i.e.*, Fe or Ti), we also conducted 2-way ANOVA on dry biomass data to explore within metal treatment responses. Nutrient data for C and N were log10 transformed to meet normality assumptions but P data met normality assumptions and data were analyzed untransformed. Pairwise multiple comparison procedures (Holm–Sidak Method) were used to determine which means were different when significant contrasts were found with ANOVA.

TRFLP profiles were used to examine the effects of nanoparticle treatment on AM fungal and rhizobia nodule communities. Detected TRFs were used as operational taxonomic units (OTU) and are treated as microbial taxa for these analyses even though each TRF may represent many microbial species [43,44,45]. The relative peak area was used for each TRF as a measure of relative abundance within the community, and peak area data were used for non-metric multidimensional scaling (NMS) analysis of community structure using PC-ORD 4 (MjM Software, Gleneden Beach, OR, USA). The Sørenson distance with a random starting configuration was used for these analyses. A maximum of 400 iterations were used for 50 runs, with data for the Monte Carlo test randomized. PC-ORD was run in autopilot mode and dimensionality confirmed manually. The best dimensionality for the order was chosen when additional dimensions did not reduce the stress by more than 5. We also used multi-response permutation procedures (MRPP), a nonparametric, multivariate procedure for testing the hypothesis of no difference between groups [46], to further confirm effects of plant and nanoparticle treatment on microbial communities. For MRPP, the Sørenson distance was used for all analyses.

## 4. Conclusions

Our examination of the effects of Fe_3_O_4_ and TiO_2_ NPs on soybean roots found that these NPs had differing effect on plant growth, nutrient content and root microbes, and that the charge of the NP played an important role in these differences. Although Fe_3_O_4_ NPs increased plant growth and leaf C content as compared to TiO_2_ NPs, leaf P levels were reduced. Moreover, negatively charged Fe_3_O_4_ NPs increased leaf P content, the translocation of Fe to leaf tissue, and decreased root colonization of rhizobia relative to positively charged Fe_3_O_4_ NPs. Although the causes of these differences with NP charge are unclear, our study suggests that plant and microbial responses to NP will be context dependent and determined by the identity of the NP, its charge and concentration.
